# Development and Characterization of Activated Carbon from Olive Pomace: Experimental Design, Kinetic and Equilibrium Studies in Nimesulide Adsorption

**DOI:** 10.3390/ma14226820

**Published:** 2021-11-12

**Authors:** Íris Nunes Raupp, Alaor Valério Filho, Aline Lemos Arim, Ana Rosa Costa Muniz, Gabriela Silveira da Rosa

**Affiliations:** 1 Graduate Program in Engineering, Federal University of Pampa, 810 Tiaraju Avenue, Alegrete 97546-550, Brazil; 2 Graduate Program in Science and Materials Engineering, Technology Development Center, Federal University of Pelotas, 1 Gomes Carneiro, Pelotas 96010-610, Brazil; alaovf@msn.com; 3 Department of Chemical Engineering, Federal University of Pampa, Av. Maria Anunciação Gomes de Godoy, 1650, Bagé 96413-172, Brazil; alinearim@unipampa.edu.br (A.L.A.); ana.muniz@unipampa.edu.br (A.R.C.M.)

**Keywords:** agro-industrial residue, adsorbent material, emerging pollutants, anti-inflammatory

## Abstract

The lack of adequate treatment for the removal of pollutants from domestic, hospital and industrial effluents has caused great environmental concern. Therefore, there is a need to develop materials that have the capacity to treat these effluents. This work aims to develop and characterize an activated charcoal from olive pomace, which is an agro-industrial residue, for adsorption of Nimesulide in liquid effluent and to evaluate the adsorption kinetics and equilibrium using experimental design. The raw material was oven dried at 105 °C for 24 h, ground, chemically activated in a ratio of 1:0.8:0.2 of olive pomace, zinc chloride and calcium hydroxide and thermally activated by pyrolysis in a reactor of stainless steel at 550 °C for 30 min. The activated carbon was characterized by Fourier Transform Infrared (FTIR) spectroscopy, X-ray Diffractometry (XRD), Brunauer, Emmett and Teller (BET) method, Thermogravimetric Analysis (TGA), Scanning Electron Microscopy (SEM), density and zero charge potential analysis. The surface area obtained was 650.9 m^2^ g^−1^. The kinetic and isothermal mathematical models that best described the adsorption were PSO and Freundlich and the highest adsorption capacity obtained was 353.27 mg g^−1^. The results obtained showed the good performance of activated carbon produced from olive pomace as an adsorbent material and demonstrated great potential for removing emerging contaminants such as Nimesulide.

## 1. Introduction

Pollution of wastewater by pharmaceuticals has generated growing concern. The emerging pollutants are harmful to the environment and human health [1]. Some countries have even banned the marketing and use of some drugs, due to their hepatotoxic effects and gastrointestinal injuries [1,2]. In the world, about 100,000 to 200,000 tons of antibiotics have been consumed and an expressive quantity of these compounds, around 30 to 90% is not entirely metabolized in human and are excreted as active compounds [3,4]. In general, the majority of drugs have high solubility in water and are not completely removed in conventional water treatment plants [1,2,5,6]. One of these contaminants is the Nimesulide, which is an anti-inflammatory and is generally prescribed to reduce fever and pain related to rheumatoid arthritis [7].

Several methods can be applied in the water treatment to removal pharmaceuticals have been reported in the literature, such as coagulation-flocculation and flotation [8], sedimentation and biotransformation, biofiltration, chemical precipitation [2,9], sand filtration [10], ozonation [9] and adsorption [1,7]. The adsorption method is one of the best processes available for the removal of pollutants from liquid effluents [11], due to the high removal efficiency, simplicity in the operation, environmental feasible and the possibility to regenerate and reuse the adsorbent after adsorption [12,13,14]. Studies have been developed using agro-industrial waste materials as low-cost adsorbents, these materials are renewable, and abundantly available [11].

One of the main agro-industrial activities of great importance in the world is the production of olive oil from olive trees [15]. Brazil has many commercial crops and the state of Rio Grande do Sul has the largest cultivated area [16], which increased from 80 ha in 2006 to 6000 ha in 2020 [17]. In the raw material processing for the production of this oil, a series of solid and liquid wastes are generated that cause serious environmental problems [11]. Among these wastes is the pomace, which consists of the pulp and core of the olive, water and residual oil [15,16,17,18]. In olive processing, an average of 20% of oil and 80% of waste is generated. Depending on the extraction system, this waste can be divided into 70% of bagasse and 10% of residual water and oil [19]. These wastes are dark-colored and are composed of high amounts of organic materials, volatile compounds and complex substances that are not easily degradable. Therefore, they are toxic to plants, decrease oxygen demand and affect the soil quality [20]. The development of new products from olive tree waste in southern Brazil has been promising due to the increase in waste generation from this activity. The soil and climate in the state of Rio Grande do Sul have specific characteristics of the region, which ensures studies in this area are still very relevant [21].

There are a variety of applications for this waste, such as energy generation through gasification, the supply of industrial ovens, agricultural use as fertilizer, animal feed, among others [22]. Alternatives have been developed for use of this waste as an adsorbent material of contaminated effluents [23], such as heavy metals, hexavalent chromium [24], cadmium [25], níquel [26], and textile dyes, safranine [27], methylene blue [28,29].

The adsorption through activated carbon from this waste has also been stood out in the removal of heavy metals [30,31] and textile dyes [32,33] from contaminated effluents. The ability of activated carbon to adsorb these pollutants from liquid effluents depends directly on the nature of the organic material used to prepare it [14].

Some research has been developed in the production of activated carbon using the olive tree stone for application as adsorbent material of pharmaceutical compounds. Especially some antibiotics, such as sulfonamide [34], tetracyclines, sulfamethazine and Amoxicillin [35]. However, a complementary approach of the use of activated carbon from olive pomace as pharmaceutical compounds adsorbent is still lacking. Literature does not report the use of activated carbon from olive pomace in the removal of Nimesulide.

The development of carbonaceous materials through chemical agents has been studied to improve their performance as adsorbents. Chemical activation enables low activation temperatures (450–700 °C), high activated carbon yield and high total surface area of produced activated carbon, which is one of the most important properties to remove pollutants from liquid effluents [36]. The application of ZnCl_2_ in chemical activation generally improves the carbon content through the formation of an aromatic graphitic structure [37]. Additionally, carbons prepared by H_3_PO_4_ activation have large particles and good sedimentation performance, which is very suitable for water treatment [38].

Thus, the development of activated carbon from olive pomace can present a great alternative for the use of agro-industrial waste, as pharmaceutical compounds adsorbent, reducing their disposal in nature. Therefore, this work aimed to develop and characterize the activated carbon from olive pomace and study the potential of adsorption of Nimesulide present in liquid effluent onto the activated carbon produced. Additionally, investigate the effects of the initial concentration, initial pH of the solutions, and dosage of adsorbent in the adsorption. Then evaluate the adsorption capacity of Nimesulide onto activated carbon as well study the equilibrium and kinetic adsorption.

## 2. Materials and Methods

### 2.1. Adsorbent Development and Reagents

**Figure 1:**
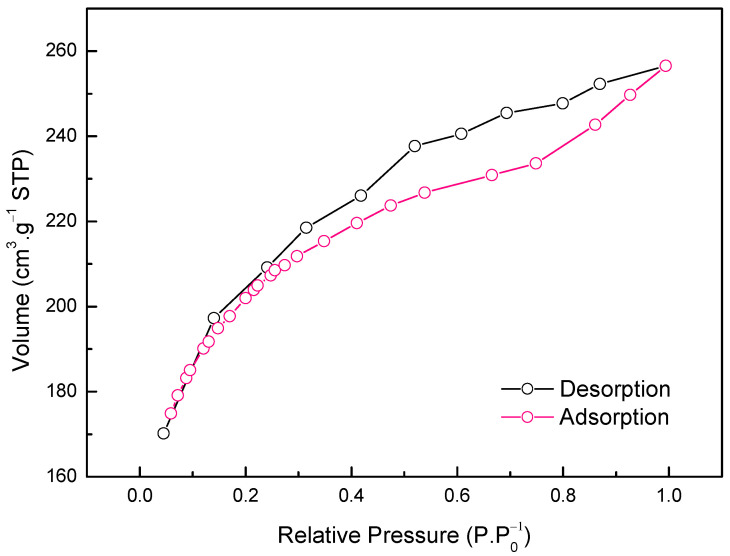
N_2_ adsorption-desorption isotherm on ACOP sample.

The raw material was obtained from the state of Rio Grande do Sul, in the south of Brazil (31°30′01.2″ S 53°30′40.4″ W). The olive pomace (OP), which consists of the pulp and stone, was oven-dried at 105 °C for 24 h (Nova Ética, model 109-1, São Paulo, Brazil). After, it was milled (Marconi, Croton model, Piracicaba, Brazil) and sieved (Bertel, model 4830, Caieiras, Brazil) to obtain particles with a diameter less than 495 nm. The OP was activated chemically in the proportion of 1:0.8:0.2 of OP, zinc chloride, and calcium hydroxide and thermally activated by pyrolysis in a stainless-steel reactor at 550 °C for 30 min (heating rate of 3 °C·min^−1^) under N_2_ atmosphere. The treated material underwent acid leaching with HCl 6 mol·L^−1^ and was washed with water until neutral and constant pH. Finally, the material was dried at 105 °C for 24 h and stored in dry conditions until utilization. This material will be referred to hereafter as ACOP. The reagents used were analytical grade ethanol, NaOH (PA), HCl (37%), and Nimesulide (Supplementary Table S1) obtained from Sigma Aldrich (São Paulo, Brazil). Figure 1 presents the molecular structure of Nimesulide. The Nimesulide stock solution was prepared with 20% ethanol for better solubilization.

### 2.2. Characterization of Adsorbent

The samples were characterized by FTIR (Perkin-Elmer UATR Two, São Paulo, Brazil), to identify the functional groups present, in the range of 500–4500 cm^−1^ with 32 scans per spectrum and 4 cm^−1^ of resolution, XRD (Rigaku ULTIMA IV, Tokyo, Japan) to analyze the crystalline and amorphous nature of the OP an ACOP, applying a Cu Kα radiation (*λ* = 1.5406 Å) at 40 kV, by scanning step over the range of 10–70° using Bragg–Brentano geometry. The density of the material was determined by a helium pycnometer (Quantachrome, Ultrapyc 1200e, Boynton Beach, FL, USA). The N_2_ adsorption-desorption isotherm was obtained by degassing the ACOP sample for 4 h under a vacuum of 300 °C and analyzed by BET method (Quantachrome Instruments, NOVA 4200e, Boynton Beach, FL, USA)), to estimate the specific surface area [39]. The thermal stability of the samples was analyzed by TGA (SHIMADZU TGA 50, Kyoto, Japan), with N_2_ flow rate of 20 mL·min^−1^, and 10 °C·min^−1^ of heating rate. The pH point of zero charge (${\mathrm{pH}}_{\mathrm{PZC}})$ was determinated with different initial pH $\left({\mathrm{pH}}_{0}\right)$ values (2–10). The pH was adjusted using 0.1 mol·L^−1^ of NaOH and HCl, as required, in 1 M NaCl solution using 1.0 g·L^−1^ of adsorbent dosage and 30 mL of the solution. The Erlenmeyer flasks containing the solutions at different pH values and the adsorbent were shaken in an acclimatized shaker at 298 K for 48 h. After, the samples were centrifuged at 3000 rpm for 10 min and analyzed in a digital pH meter (Metrohm, 827 pH Lab, São Paulo, Brazil) to verify the final pH $\left({\mathrm{pH}}_{f}\right)$ values of the aqueous solution. The adsorbent surface morphology was analyzed by SEM (Hitachi TM-3000, Tokyo, Japan) before and after the Nimesulide adsorption onto ACOP. The micrographs were analyzed in magnifications of 500× and 1000×.

### 2.3. Adsorption Experiments

From preliminary trials, were obtained operating conditions for the experiments. All the adsorption experiments were conducted in batch using a synthetic solution in different concentrations, which was kept under agitation at 150 rpm in a shaker (NOVA ÉTICA, model 109-1, São Paulo, Brazil). The tests were performed in duplicate. In the experiments, the liquid phase was separated from the adsorbent by centrifugation at 4000 rpm for 10 min. The Nimesulide residual concentration was quantified using a standard curve with concentration range from 1 to 50 mg·L^−1^ at a maximum wavelength of 392 nm using a UV/Visible spectrophotometer (EQUILAM, UV 755B, Diadema, Brazil)). The adsorption capacity, Q (mg·g^−1^) and the efficiency of removal, E (%) by ACOP are represented by Equations (1) and (2), respectively.

(1) $$Q=\frac{V\left(C_{0}-C_{e}\right)}{m}$$

(2) $$E=\frac{C_{o}-C_{e}}{C_{o}}\cdot 100\%$$

where $C_{0}$ and $C_{e}$ are the initial and equilibrium Nimesulide concentrations (mg L^–1^), respectively, V is the volume of the solution (L), and m is the mass of biosorbent used (g).

The experimental design technique was applied in order to investigate the influence of operational conditions in Q and E of the Nimesulide adsorption onto ACOP. The factorial design (FD) was implemented to obtain the effect of three factors using as independent variable, such as the initial pH of the solution (pH), adsorbent dosage of ACOP $\left(A_{d}\right)$ and initial concentration of the solution $\left(C_{0}\right)$. The FD was based on a 2^3^ experimental design with 3 central points and the experiments were carried out during 3 h with replicates. To evaluate the significance of the model, 95% of confidence interval (*p*_value ≤ 0.05) was utilized. The levels tested for each factor are presented in Table 1.

### 2.4. Adsorption Kinetic and Isotherm Models

The kinetic experiments were carried out by adding the solution containing 30 mL of Nimesulide solution in erlenmeyer flasks with ACOP. The samples were taken at preset time intervals (2 to 360 min). The experimental data were adjusted to the different kinetic models of Pseudo-First Order (PFO) [40], Pseudo-Second Order (PSO) [41], and Elovich [42], which are represented by Equations (3)–(5), respectively. To identify the mass transfer steps, the Weber–Morris model was used which is represented by Equation (6) [43].

(3) $$q_{t}=q_{e}\left(1-\exp^{\left(-k_{1}t\right)}\right)$$

(4) $$q_{t}=\frac{{q_{e}}^{2}\left(k_{s}t\right)}{\left(1+q_{e}k_{s}t\right)}$$

(5) $$q_{t}=\frac{1}{b}\ln\left(1+\mathrm{abt}\right)$$

(6) $$q_{t}=k_{\mathrm{int}}t^{1/2}+C$$

where t is the time (min), $q_{t}$ is the amount adsorbed (mg·g^–1^) at the time t, $(k_{1}$) is the pseudo-first-order rate constant (min^–1^), $k_{s}$ is the pseudo-second-order rate constant (g· mg^–1^·min^–1^), a is initial velocity due to Elovich model (mg·g^−1^·min^−1^), b is the desorption constant of the Elovich model (g mg^−1^), $k_{\mathrm{int}}$ is the rate constant for intraparticle diffusion (mg·g^–1^·min^–0.5^) and C (mg·g^–1^) is a constant that accounts for the thickness of the boundary layer.

Equilibrium isotherms were elaborated, varying the initial concentration of the solution from 15 to 500 mg·L^−1^. The experiments were conducted until reaching equilibrium. The isotherm models, relating to adsorption equilibrium studied were the Langmuir [44] and Freundlich [45], represented by Equations (7) and (8), respectively.

(7) $$q_{e}=\frac{q_{m}K_{L}C_{e}}{1+\left(K_{L}C_{e}\right)}$$

(8) $$q_{e}=K_{F}C_{e}^{\frac{1}{n_{F}}}$$

where $q_{m}$ is the maximum adsorption capacity (mg·g^−1^) and $K_{L}$ is the Langmuir constant (L·mg^−1^); $K_{F}$ is the Freundlich constant (mg·g^−1^)(mg·L−1)−1nF and $\frac{1}{n_{F}}$ is the equilibrium constant indicative of adsorption intensity and associated to the heterogeneity of the adsorbent surface. The adjustments of the mathematical models to the experimental data were evaluated by the correlation coefficient ($R^{2}$) and average relative error (ARE) represented by Equations (9) and (10) respectively.

(9) $$R^{2}=\left(\frac{\sum_{i}^{n}q_{i,\exp}-{\bar{q}}_{i,\exp}^{2}-\sum_{i}^{n}q_{i,\exp}-q_{i,\mathrm{model}}^{2}}{\sum_{i}^{n}q_{i,\exp}-{\;q\;¯}_{i,\exp}^{2}}\right)$$

(10) $$\mathrm{ARE}=\frac{100}{n}\sum_{1}^{n}\left|\frac{q_{i,\mathrm{model}}-{\;q}_{i,\exp}}{q_{i,\exp}}\right|$$

where $q_{i,\exp}$ is the experimental values of adsorption capacity obtained, ${\bar{q}}_{i,\exp}^{2}$ is de average of each adsorption capacity measured, $q_{i,\mathrm{model}}$ is the predicted values obtained by the fitted model, and n is the number of experimental data.

## 3. Results and Discussion

### 3.1. Characterization of OP and ACOP

The helium density of ACOP obtained was 1.5148 g·cm^−3^. The literature reports values between 2.2 and 3.3 g·cm^−3^ [13,46]. The low value obtained can indicate the presence of hollow particles, that is a kind of powder contained interior hollow structure. This hollow structure is usually covered by a solid shell, meaning there are empty spaces inside the particles [47,48].

Figure 1 shows the characteristics of ACOP determined by $N_{2}$ adsorption-desorption. It was possible to identify the type of pores present in the solid by evaluating the isotherm curve. The results indicated that the isotherm obtained is type IVa according to IUPAC classification, which indicates the predominance of mesoporous particle size distribution. The presence of the type H3 hysteresis loop characterizes the slit-shaped pores [49,50].

The BET surface area ($S_{\mathrm{BET}})$ obtained were 650.9 m^2^·g^−1^. The physical properties obtained for the ACOP are comparable to the literature for the activated carbon from olive bagasse. Table 2 presents the parameters used by the literature and for this work to obtain the activated carbon from olive wastes. Baçaoui et al. [51] reported 514 m^2^·g^−1^ for surface area with 800 °C of activation temperature (pyrolysis stage) during 30 min. Demiral et al. [52] studied the influence of the activate temperature and time and values between 523 and 617 m^2^·g^−1^ were obtained for the surface area with 750 °C activation temperature during 30–60 min, respectively. Setting the activation time to 30 min, values between 523 and 947 with 750–900 °C were obtained, respectively. The behavior of the results obtained by Demiral et al. [52] showed that the increase in the activate temperature and time increases the pores in the material and forms new pores by devolatilization and carbon burn-off due to the C–H_2_O reactions, which indicates the burn-off of the activated carbon is a very important effect. Al-Ghouti; Sweleh [13] studied the activated carbon prepared from green olive stones at 500 °C for 3 h and found a surface area of 9.11 m^2^·g^−1^ and 0.151 cm^3^·g^−1^ of the pore volume. The low value obtained for the surface area can be explained by the very long time of activation, which can induce the destruction of high porosity by external ablation of carbon particles instead of the development and widening of microporosity [52]. The results obtained were satisfactory, with a higher surface area at lower activate temperature and time conditions than those reported in the literature, justifying the very attractive properties of the material obtained, decreasing the energy cost in the pyrolysis stage, knowing that the adsorbent material is responsible for about 70% of the operational costs of adsorption [53,54].

**Figure 2:**
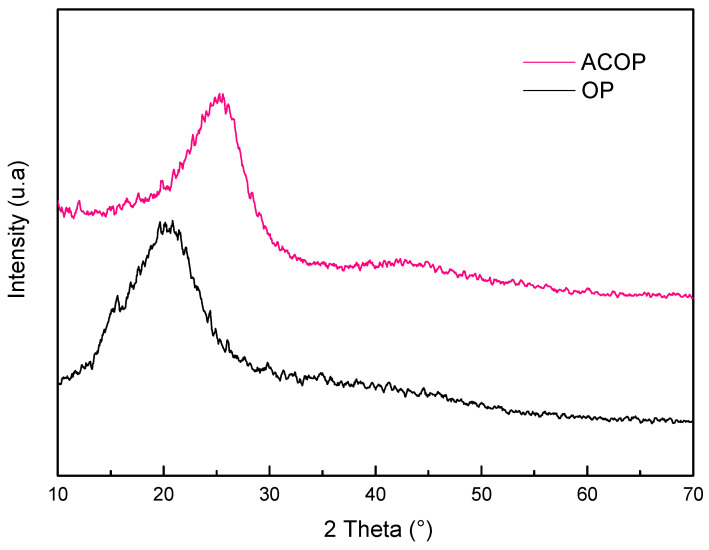
X-ray diffractograms of OP and ACOP samples.

Figure 2 presents the X-ray diffractograms of OP and ACOP. It is possible to identify typical crystalline structures of cellulose in OP, located at 2θ 15.65°, 20.85°, 34.85° corresponding to crystallo-graphic plane (1 0), (0 2 1) and (0 0 4) of cellulose I [55,56,57]. The ACOP diffractogram indicated a typicacl amorphous carbon and showed peaks at 2θ 25.25° and 43°, that corresponds to the (0 0 2) and (1 0 0) plane, respectively, which are graphite-like reflections indicating the graphitic ordering in molecular planes [58,59,60,61]. No residues of the chemical activating agents were identified in the diffraction measurements, which indicates that the acid washing step was efficient to remove any residual inorganic material which could be present on the carbon material surface [39,62].

**Figure 3:**
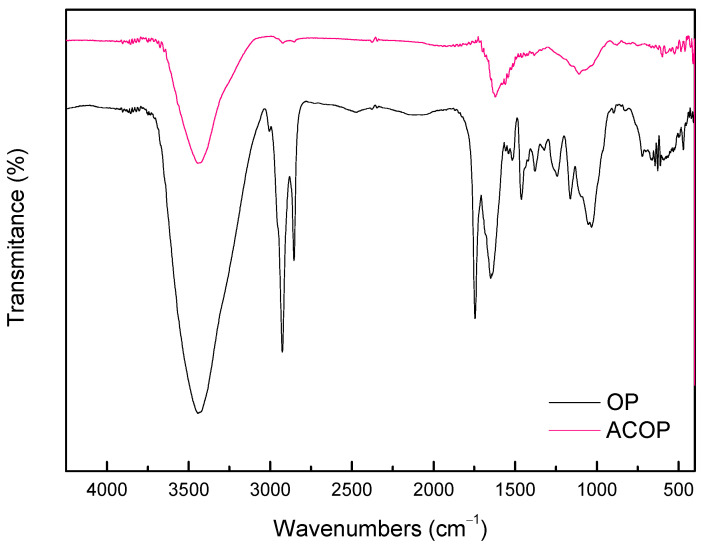
FTIR vibrational spectra of OP and ACOP.

The FTIR analysis of the OP and ACOP samples are presented in Figure 3. Could be inferred that both FTIR spectra showed similar bands. However, the intensity in ACOP was reduced when compared to OP. The band around 3435 cm^−1^ represents the O–H stretching mode which corresponds to hydroxyl groups [33,34,52]. The band between 2925 and 2853 cm^−1^ in OP analysis, which were not observed in ACOP, corresponds to C–H bands of methyl and methylene groups existing in cellulosic material, and this band indicates the presence of various aminoacids. The stretching vibrations between 1745 and 1641 cm^−1^ are usually assigned to C=O of ketones, aldehydes, lactones or carboxyl groups [33]. Further, the stretching between 1000 and 1250 cm^−1^ usually corresponds to oxidized carbons C–O stretching in acids, alcohols, phenols, ethers and ester groups [33,52]. The infrared spectroscopy provided information about the chemical structure of the OP and ACOP. The presence of groups such as hydroxyl and carboxyl, ethers and aromatic compounds indicates the lignocellulosic structure properties of olive wastes [33].

**Figure 4:**
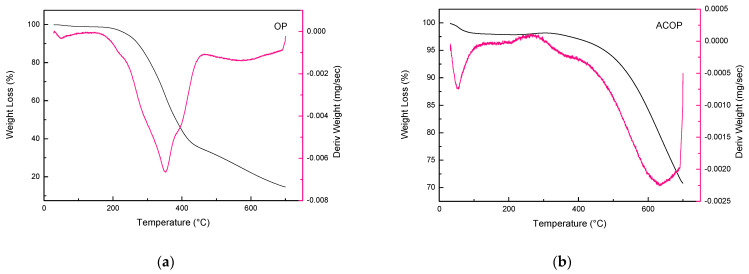
TG and DTG curves of OP (**a**) and ACOP (**b**).

The thermogravimetric curves for the OP and ACOP are presented in Figure 4a,b, respectively. These curves demonstrated the thermal stability of the materials over a range of temperatures (from 25 to 700 °C). The TG and DTG curves to OP showed weight losses in three different temperature ranges. The first weight loss (from 28 and 146 °C) represents 1.15% of the initial weight is due to evaporation of free water in the samples [63,64]. The second weight loss (from 146 to 463 °C) represents 64.44% of the initial weight, is probably due to hemicellulose and cellulose degradation. The third weight loss (from 463 to 691 °C) represents 19.28% of the initial weight loss, corresponds to lignin decomposition [64,65,66,67]. Lignin decomposition starts at low temperatures (similar to hemicellulose) and continues up to 600 °C [68]. Thus, the tailing at the end of the curve is considered the decomposition of lignin, to be the most complex and stable of the material components [69]. The TG and DTG curves to ACOP showed greater thermal stability compared to OP. The first weight loss (from 32 to 126 °C) represents 1.95% of the initial weight, representing water evaporation. The weight loss between 265 and 410 represents 1.13% of the initial weight loss and characterizes the pyrolytic decomposition of hemicellulose, cellulose and lignin residue. And the most representative weight loss occurred between 410 and 691 °C, which corresponds to 25.22% of the initial weight loss and represents a rigid carbon skeleton [69].

**Figure 5:**
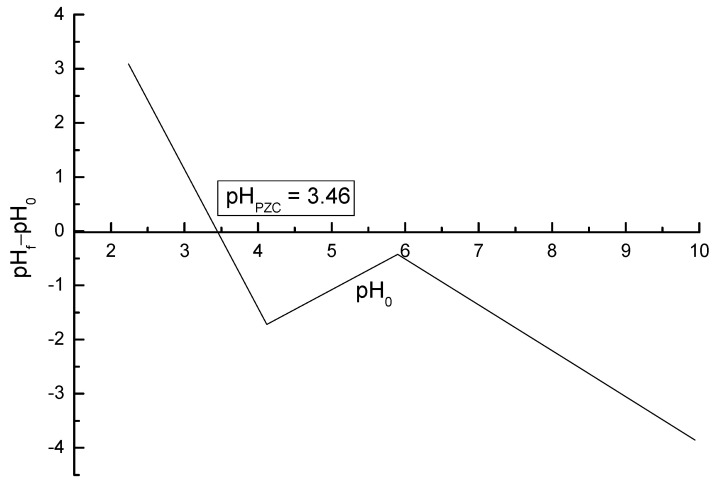
ACOP ${\mathrm{pH}}_{\mathrm{PZC}}$.

The surface charge of the adsorbent has greatly influence in its adsorption capacity. The ${\mathrm{pH}}_{\mathrm{PZC}}$ analysis allows the prediction of the surface charge of the adsorbent as a function of the pH and indicates the pH value in which the charge of the surface is zero. The surface charge for ACOP as a function of pH is shown in Figure 5. The ${\mathrm{pH}}_{\mathrm{PZC}}$ value obtained was 3.46, which indicates the acidic nature of the ACOP surface. When the pH of the solution in contact with the adsorbent is lower than 3.46, the adsorbent surface is supposed to be positively charged, where the functional groups are protonated. This behavior favors the anionic species adsorption. In contrast, when the pH of the solution is higher than 3.46, the adsorbent has a negative charge surface, which favors the cationic species adsorption. The value obtained for the ${\mathrm{pH}}_{\mathrm{PZC}}$ indicates that ACOP has a negatively charged surface. The pH range studied in the experiments for Nimesulide adsorption was higher than 3.46 (8–11). This result may suggest that Nimesulide has a cationic character. In this case, the functional groups release H^+^ [1,70].

### 3.2. Experimental Design and Statistical Analysis

**Figure 6:**
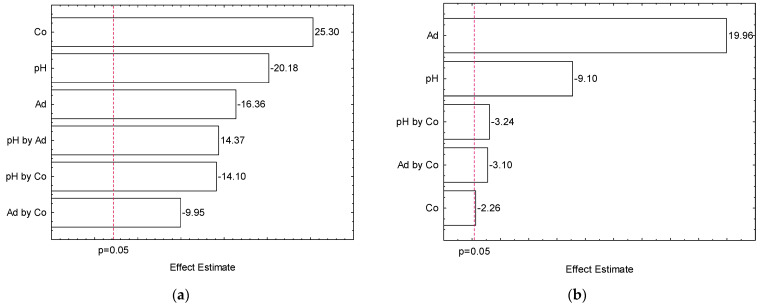
Pareto chart of estimated effects on Q (**a**) and E (**b**).

The adsorption experiments were performed according to the experimental matrix presented in Table 3. The pareto chart presented in Figure 6 shows the estimated effects on the adsorption process of Nimesulide for adsorption capacity (a) and removal efficiency (b).

The highest adsorption capacity was obtained with the lowest adsorbent dosage and higher initial concentration at pH 8 (run 5). For the removal efficiency, the highest value was obtained utilizing a higher adsorbent dosage and the lowest initial concentration at pH 8 (run 3). Figure 6a,b shows the Pareto plot of the standardized effects at *p* = 0.05. Analyzing these results, it can be concluded that that all the parameters presented significant effects on the adsorption capacity and removal efficiency of Nimesulide by AOCP. In Figure 6a the greatest significant effect was the initial concentration of the solution. This effect is associated to a greater driving force for the diffusion of the Nimesulide within the ACOP particles [71]. The second and third most significant effects were the pH of the solution and the adsorbent dosage, respectively, with a negative influence on the process. In the process of adsorption the pH of the solution is one of the most important factors, because it affects the surface charge (protonation or deprotonation) of the adsorbent [71,72].

As suggested by the Pareto chart results, the dependence between the pH and the adsorbent performance is negative, which means that the adsorption capacity increase with the decrease of pH. According to ${\mathrm{pH}}_{\mathrm{PZC}}$ in Figure 5, ACOP has negative surface charge with a solution pH greater than 3.46. Thereby, the ACOP features negative surface charge in pH 8, increasing the interaction with cationic solutions. However, for acidic pH ranges below 8, Nimesulide precipitates, making adsorption difficult, so the studies were conducted at basic pH values. Additionally, the decrease of adsorbent dosage may indicate that for the highest values of adsorbent dosage studied, the ACOP has not reached saturation [73]. In Figure 6b the more pronounced effect was the adsorbent dosage. This can be explained by the fact that with the increase in the adsorbent dosage, there is also an increase in the number of available sites for adsorption. Thus, the removal efficiency is higher while the adsorptive capacity of the material decreases. The second expressive effect was the pH of the solution, with a negative influence on the process as also presented in Figure 6a.

It was chosen as an adequate condition to perform the adsorption kinetic and equilibrium studies the conditions of run five, which presents pH 8 and a concentration of 30 mg·L^−1^ for the solution of Nimesulide and 0.1 g·L^−1^ for the dosage of adsorbent. At this condition the best values for the adsorptive capacity using less amount of adsorbent material were obtained.

### 3.3. Adsorption Kinetics

**Figure 7:**
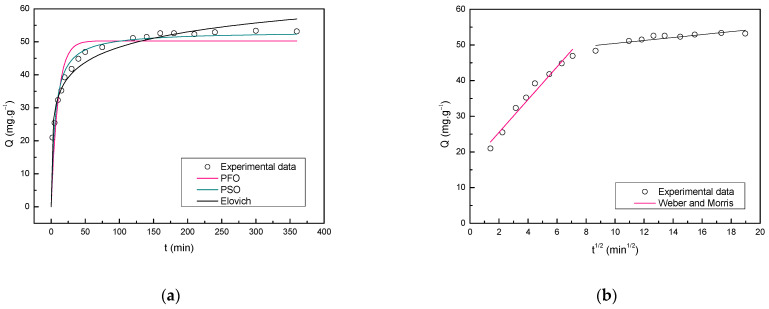
Kinetic curves (**a**) and intraparticle diffusion model (**b**) of Nimesulide adsorption onto ACOP.

The kinetic curves of Nimesulide adsorption onto ACOP for the PFO, PSO and Elovich models are presented in Figure 7a. The intraparticle diffusion model by Weber and Morris equation is presented in Figure 7b. The parameters obtained in the adjustment of the experimental data to mathematical models are shown in Table 4.

In Figure 7a, could be observed that higher sorption rates were obtained in the beginning of the experiments due to the greater number of available sites in the ACOP surface. As the contact time increases, the adsorption rate tends to decrease, this profile indicates that the external surface adsorption occurred quickly [74]. This behavior may suggest that the fast first step is limited by diffusion and followed by a slower second step which is limited by diffusion in smaller pores, characterized by slow adsorption [75,76]. The experimental data showed that a great amount of Nimesulide was removed within 50 min and the equilibrium was reached at about 120 min. According to Table 3, could be considered that all models fitted well with the experimental data. According to the correlation coefficient ($R^{2}$) and average relative error (ARE) obtained for the models, the kinetic model that best described the adsorption of Nimesulide onto ACOP was the Elovich followed by the PSO and PFO model, respectively. The Elovich model presented a $R^{2}$ of 0.975 and ARE of 1.509. Additionally, presented a value of 94.696 mg·g^−1^·min^−1^ for the initial adsorption rate (a) and 0.150 g·mg^−1^ for the desorption constant (b). This model suggests that the adsorption process is chemical (chemisorption) [77].

However, in some situations, the model with the highest $R^{2}$ may presents failures in the adjustment, characterized by a tendency to overestimate for low equilibrium concentrations. The residue scatter plot (Supplementary Figure S3) shows random scattering of residues along the x axis. Analyzing the global behavior of the models (Supplementary Figures S1–S3), the PSO model was the one that best fitted the experimental data, presenting minimal deviation and a satisfactory distribution. It could be assumed that the non-linear PSO equation is reliable to make a relation between studied conditions and considered variables in the removal of Nimesulide presenting 0.968 and 1.741 for $R^{2}$ and ARE, respectively.

Contrary to the PFO model that applies only to a certain period of adsorption, the PSO model predicts the behavior over the whole range of adsorption studies and the rate-controlling step is chemisorption. The Weber–Morris equation was used to evaluate the diffusion on adsorption kinetic. The literature reports that, generally, when the straight line of Weber and Morris passes through the origin, the intraparticle diffusion is the only rate limiting step of the process. However, if the straight-line does not pass through the origin, it can be considered that the process is controlled by other mechanisms, such as external/film diffusion [78,79,80,81]. The plot of $Q\;\mathrm{vs}.{\;t}^{1/2}$ presented in Figure 7b shows multi-linearity, where two steps in the adsorption process can be seen. The adjustment of the model in the experimental data did not pass through the origin, suggesting that the adsorption process is controlled by external and intraparticle diffusion in the first step. The second step corresponds to the final equilibrium stage. The model predicted 12.495 mg·g^−1^ for C, which represents the thickness of the boundary layer, and 5986 g·mg^−1^·min^−1/2^ for $K_{\mathrm{int}}$, which is the internal diffusion constant.

### 3.4. Adsorption Isotherms

**Figure 8:**
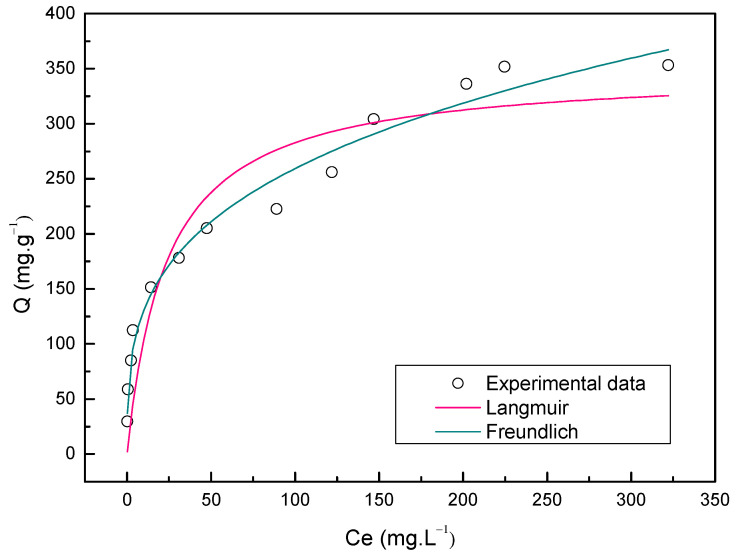
Equilibrium isotherms of Nimesulide adsorption onto ACOP.

The experimental and calculated equilibrium data concerning the sorption of Nimesulide onto ACOP are depicted in Figure 8. The isotherms models of Langmuir and Freundlich were fitted to experimental data. The parameters of the isotherms were estimated using the nonlinear regression method, obtained in the adjustment of the experimental data to mathematical models and are summarized in Table 5.

The adsorption isotherms of Nimesulide onto ACOP are shown in Figure 8 and indicate that the isotherm profiles were favorable [82]. The equilibrium data of Nimesulide sorption onto ACOP can be better described by the Freundlich model with the higher value to $R^{2}$ (0.955) and the lower ARE value (7.198%) as indicated in Table 5. The Freundlich model describes a heterogeneous multilayer adsorption [83]. The $k_{F}$ constant (63.689 (mg·g−1)(mg·L−1)−1nF) represents the Freundlich constant and 1/$n_{F}$ (0.294), the adsorption intensity. The 1/$n_{F}$ value, which is between 0 and 1 indicate that the adsorption of Nimesulide onto ACOP is favorable, and is related to heterogeneous interaction and to the chemical nature of the process [34]. Table 6 presents the adsorption capacity obtained for the ACOP and compare with the values presented in the literature researches for ACOP and Activated Carbon from Olive Stone (ACOS). The highest maximum adsorption capacity (q_max_) was obtained by ACOP for the adsorption of Nimesulide with a value of 353.27 mg·g^−1^. The value for the maximum adsorption capacity obtained for the present study was higher than the values reported in the literature for activated carbons produced from olive wastes with similar chemical activation methods. This result indicates that ACOP produced through chemical activation is an effective adsorbent in removing Nimesulide from aqueous solutions.

**Figure 9:**
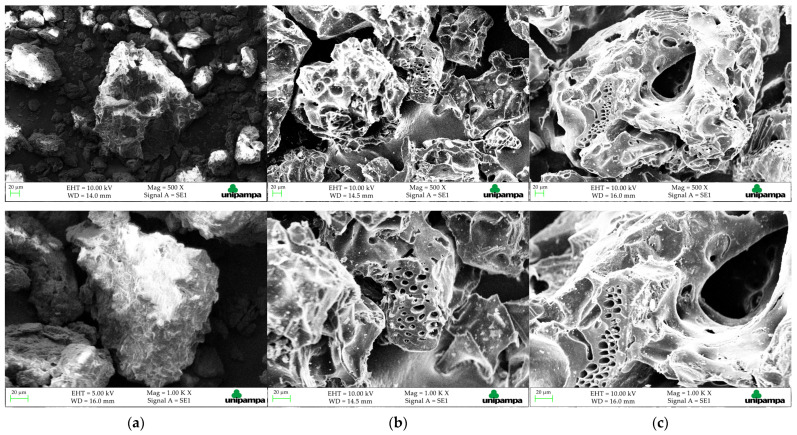
SEM images: (**a**) OP, (**b**) ACOP and (**c**) ACOPN.

The micrographs presented in Figure 9a for the OP shows complex surface structures with an uneven and rough texture. Additionally, was not possible to identify available pores on the surface. In contrast, SEM images of ACOP in Figure 9b shows changes in the morphological structure of the solid surface and pore distribution attributed to the chemical and physical treatment. The ACOP exhibited a heterogeneous surface. Furthermore, a large amount of pores with different sizes and shapes were observed, and were well developed. The pore size showed random distribution and can be compared to a sponge-like structure. SEM images in Figure 9c shown no significant changes in the morphology and structure of ACOPN since the adsorption process should not modify the morphology of the material [84]. Therefore, the solid pores did not show any obstruction, which suggests its good performance as an adsorbent material.

## 4. Conclusions

The production of activated carbon by the pyrolysis process proved to be efficient, ensuring attractive properties to the material, which presented a $S_{\mathrm{BET}}$ of 650.9 m^2^·g^−1^. The pore size classification is mesoporous. These properties ensured the potential for its use in the removal of Nimesulide. The experimental design allowed the evaluation of the parameters involved in the adsorption process, such as the initial concentration, pH of the solution containing the contaminant, and dosage of the adsorbent. The maximum adsorption capacity obtained experimentally was 353.27 mg·g^−1^, and the kinetic and isotherm mathematical models that best fitted the experimental data were PSO and Freundlich, respectively.

## Figures and Tables

**Table 1:** Real and coded values of the experimental design.

Factors	Levels		
	−1	0	1
pH	8	9.5	11
A_d_ (g·L^−1^)	0.1	0.3	0.5
C_0_ (mg·L^−1^)	10	20	30

**Table 2:** Parameters used to obtain the activated carbon from olive wastes.

	Present Study	Baçaoui et al. [51]	Demiral et al. [52]		Al-Ghouti; Sweleh [13]
T (°C)	550	800	750	750–900	500
t (min)	30	30	30–60	30	3
S_BET_ (m^2^·g^−1^)	650.9	514	523–617	523–947	9.11

**Table 3:** Matrix of the experimental design and the corresponding responses.

run	pH	A_d_ (g·L^−1^)	C_0_ (mg·L^−1^)	Q (mg·g^−1^)	E (%)
1	8 (−1)	0.1 (−1)	10 (−1)	38.59 ± 3.88	37.31 ± 3.75
2	11 (+1)	0.1 (−1)	10 (−1)	19.65 ± 1.92	20.31 ± 1.99
3	8 (−1)	0.5 (+1)	10 (−1)	19.08 ± 0.22	95.70 ± 0.77
4	11 (+1)	0.5 (+1)	10 (−1)	16.53 ± 0.18	82.12 ± 0.88
5	8 (−1)	0.1 (−1)	30 (+1)	142.98 ± 6.78	48.49 ± 3.09
6	11 (+1)	0.1 (−1)	30 (+1)	39.84 ± 0.11	13.50 ± 0.26
7	8 (−1)	0.5 (+1)	30 (+1)	53.93 ± 0.20	89.59 ± 0.04
8	11 (+1)	0.5 (+1)	30 (+1)	35.92 ± 0.75	60.26 ± 1.25
9 (C)	9.5 (0)	0.3 (0)	20 (0)	9.72 ± 1.61	29.03 ± 4.97
10 (C)	9.5 (0)	0.3 (0)	20 (0)	10.24 ± 1.68	30.57 ± 5.19
11 (C)	9.5 (0)	0.3 (0)	20 (0)	12.10 ± 2.69	36.31 ± 8.06

**Table 4:** Kinetic parameters for the Nimesulide adsorption onto ACOP.

PFO		PSO		Elovich		Weber and Morris	
q_1_ (mg·g^−1^)	50.277	q_2_ (mg·g^−1^)	53.136	a (mg·g^−1^·min^−1^)	94.696	K_int_ (g·mg^−1^·min^−1/2^)	4.594
K_1_ (L·mg^−1^)	0.101	K_2_ (L·mg^−1^)	0.003	b (g·mg^−1^)	0.150	C (mg·g^−1^)	16.304
R^2^ (%)	0.905	R^2^ (%)	0.968	R^2^ (%)	0.975	R^2^ (%)	0.932
ARE (%)	9.619	ARE (%)	1.741	ARE (%)	1.509	ARE (%)	5.103

**Table 5:** Equilibrium isotherm parameters for the Nimesulide adsorption onto ACOP.

Langmuir		Freundlich	
$q_{\max}$ (mg·g^−1^)	348.533	$k_{F}$ (mg·g^−1^)(mg·L−1)−1nF	67.237
$k_{L}$ (L·mg^−1^)	0.043	1/$n_{F}$	0.294
$R^{2}\;\left(\%\right)$	0.855	$R^{2}\;\left(\%\right)$	0.955
ARE (%)	24.461	ARE (%)	7.198

**Table 6:** Adsorption capacity obtained for the ACOP and literature researches.

Adsorbent	Activating Agents	Adsorption Capacity (mg·g^−1^)	Contaminant	Reference
ACOP	ZnCl_2_/H_3_PO_4_	353.27	Nimesulide	Present study
ACOP	KOH	66.22	Sulfadiazine	[34]
ACOS	ZnCl_2_	42.01	Tetracycline	[35]
ACOS	H_3_PO_4_	186.0	Tetracycline	[35]

## Supplementary Materials

The following are available online at https://www.mdpi.com/article/10.3390/ma14226820/s1, Figure S1: Residuals versus the predicted plot for PFO model, Figure S2: Residuals versus the predicted plot for PSO model, Figure S3: Residuals versus the predicted plot for Elovich model, Table S1: Nimesulide molecular structure and physical chemical properties.
