# Supplementary material for: Development and Characterization of Activated Carbon from Olive Pomace: Experimental Design, Kinetic and Equilibrium Studies in Nimesulide Adsorption
Source: Materials (Basel). 2021 Nov 12;14(22):6820. doi: 10.3390/ma14226820 (PMC8622804; doi:10.3390/ma14226820)
Supplement: Supplementary file 1 [file materials-14-06820-s001.zip › materials-1373328-supplementary.pdf]

Table S1. Nimesulide molecular structure and physical chemical properties.

**Parameter**	**Character/Value**
Molecular structure	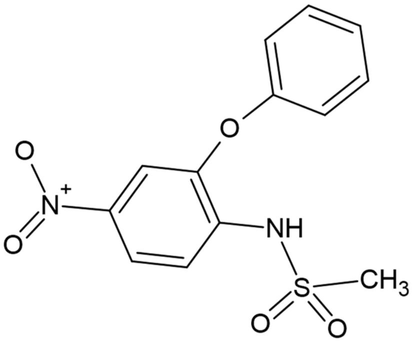
Molecular formula	$C_{13}H_{12}N_2O_5S$
Molecular weight (g mol^{-1})	308,311
Wavelength λ_{max} (nm)	392

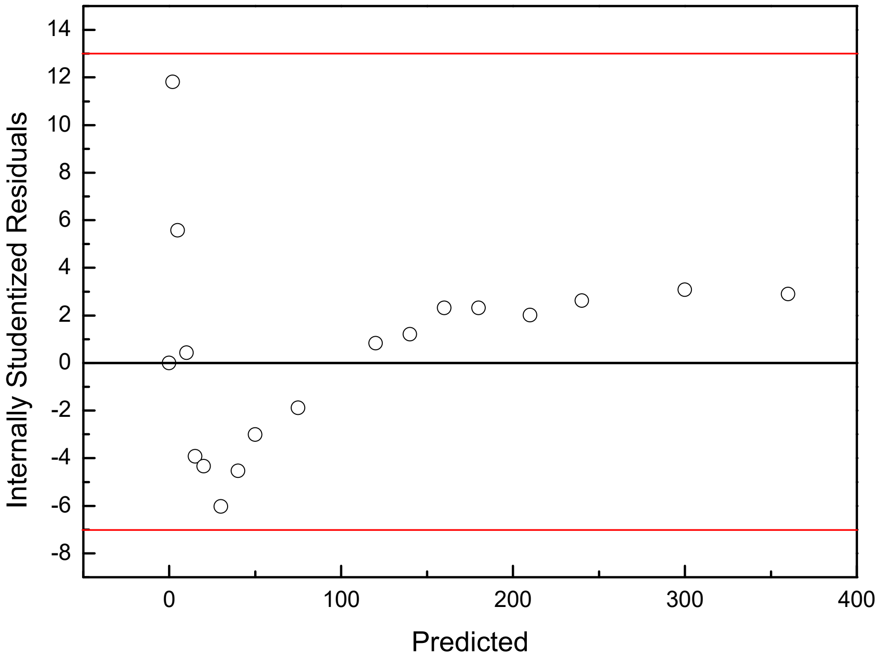

Figure S1. Residuals versus the predicted plot for PFO model.

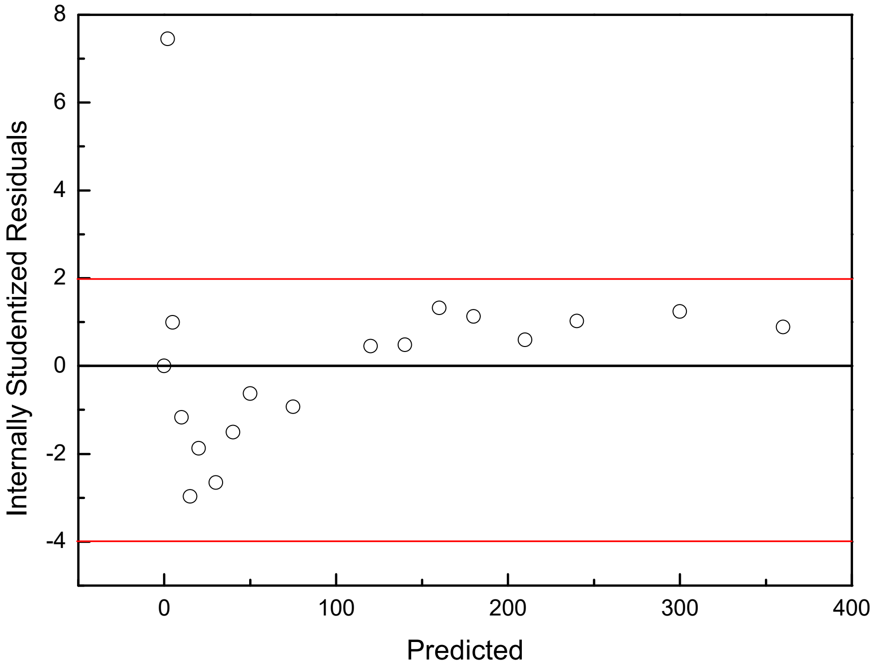

Figure S2. Residuals versus the predicted plot for PSO model.

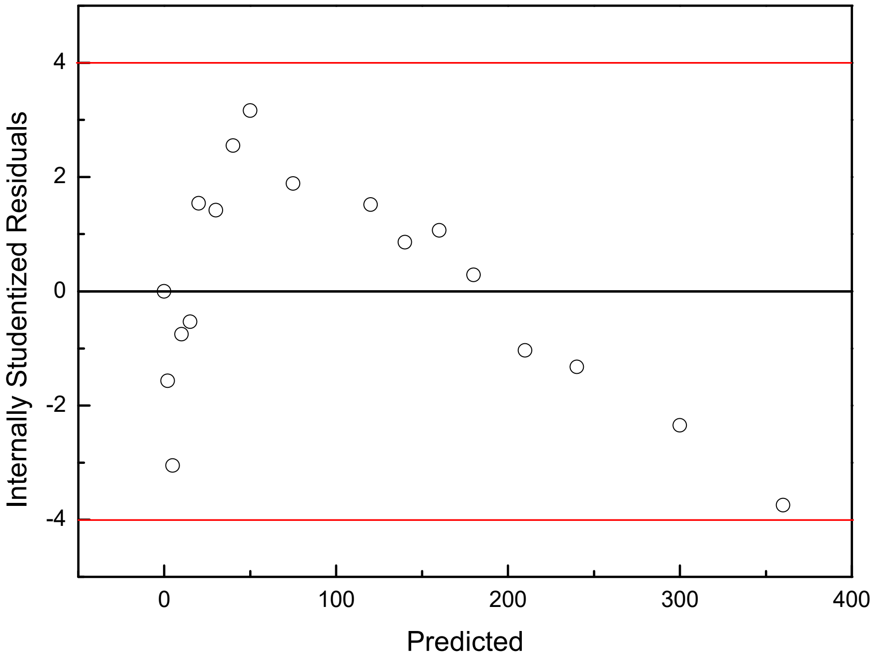

Figure S3. Residuals versus the predicted plot for Elovich model.
